# Annotations

**Published:** 1900-07-07

**Authors:** 


### Annotations.

### The Midwives Bill.

The end of the Midwives Bill came last week, for it is neither to be hoped nor to be expected that it will get another chance this session. It was a curious finish; the Bill being talked out by its own promoter. Nevertheless, we can but think that the wide discussion which the proposal has secured for itself this year, and the ease with which the Bill went through its earlier stages, will have a marked influence upon any measure which may be introduced in any future session. What the measure which now lies dead has done has been to impress people with the reality of the demand for legislation. It is to be hoped, however, that the position taken by this Bill, and the somewhat unexpected interest shown in it, may induce the Government to take the matter by the hand in another session. It would in many ways be better that legislation on so important a subject, and one so intimately interlocked with other interests, should be formulated by those who will consult and act in accordance with the advice of bodies already established by law—so that the whole of the law regarding the practice of medicine and the ancillary arts, and perhaps even the ancillary trades, should be arranged as a co-ordinate whole—than that each bit of law making should stand independent of, or possibly antagonistic to the rest. It is pretty clear by now what sort of a Bill the General Medical Council will be willing to approve of, and if the Government were to take that as the basis, they could easily pass a useful measure which would remove a great scandal.

### Competing Sanitary Authorities.

Among the subjects which came up for discussion at the last meeting of the School Board for London was one of very great sanitary importance, the School Management Committee having presented a report recommending the adoption of certain measures for the prevention of the spread of infectious diseases among the children attending their schools. The committee recommend, among other things, that as an experiment for two years a system of "preventive visitation" should be instituted in certain districts, and that for this purpose the Board should authorise the engagement of two medical men at a salary of £250 per annum each, and six nurses at a salary of £80 per annum each, one medical man and three nurses being allotted to each of the sets of schools selected. In regard to the desirability of some such system of medical inspection, not merely of schools and their sanitary condition but of the children attending the schools, and in case of necessity of the homes from which these children come, we can entertain no shadow of doubt; but the proposal to establish a sanitary network, which if it is to be of real utility must extend over the whole of the metropolis, and must at every turn come in touch with if it do not actually compete with the legally constituted sanitary service is another matter, and it is a serious question whether it would be wise to encourage the establishment of such a duplicate sanitary service as would be involved in the suggestion now under discussion. The ideal would be to concentrate these inspectorial functions rather than to break them up among many authorities, each working for what is in reality only a small part of the great subject of public health.

### The Bloemfontein Scandals.

The week has been full of discussions regarding the treatment of the sick in South Africa, the disclosures made by Mr. Burdett-Coutts having become the text not only for a full debate in Parliament, but for letters and articles innumerable in the daily papers; and there is some danger lest the real point should be lost sight of, namely, the question, Who is responsible? There is plenty of evidence as to the ample supplies, the admirable treatment, the comforts, and even the luxuries which were provided for the sick at certain times and at certain places. It need hardly be pointed out, however, that much of this evidence is quite beside the mark. The question is not whether there has been any special or serious failure in the hospital arrangements in places where supplies were plentiful and the hospitals were fully staffed; but whether, when enormous numbers of fever-stricken men were poured into hospitals situated at a distance from the base, and far too small to receive them all, a proper and due proportion of the available transport was employed in bringing up doctors, orderlies, nurses, and material, so as to make the hospitals even approximately equal to the demands upon them. Mr. Burdett-Coutts makes many other charges, especially in regard to the cast-iron rules by which all army organisation is dominated, but the central charge is that of neglect to furnish for the medical side a reasonable and sufficient share of the transport to enable the sick to be properly dealt with. Upwards of a thousands tons a day of stuff of one kind and another were being poured into Bloemfontein; newspaper correspondents and "innumerable attendants and nondescript people" were coming up; and it is difficult to understand why the transport authorities were permitted to keep back the medical supplies, and why the whole resources of Bloemfontein were not requisitioned to tide over the emergency. It is an old, old difficulty. People in this country, who trusted that all was being done for the sick in South Africa, will certainly want to know what sort of stores were being sent up the line at the time when, according to the statements now made, it was impossible in view of the difficulties of transport to send up the necessary medical supplies, and what sort of people were going up and down the line at the time when, owing to the pressure of work thrown upon the railway, it was also found impossible to send medical men and nurses to the front. Who is responsible for passing forward newspaper correspondents and "nondescript people" when doctors were kept back, and for allowing the trucks to be filled with general stores, champagne probably among the rest, when it was "impossible" to forward the tents, or even the blankets, which, according to Mr. Wyndham's statement, had been sent to the Cape in such profusion? The whole things seems to resolve itself into one of the relations existing between the medical and the military authorities, and we cannot but fear that much of the resulting muddle may be traced back to the remains of those old traditions of the War Office, which teach a doctor to keep his proper place, and, above all things, not to make himself troublesome. The real question on which the public would like to be informed is, Did the doctor keep his "proper place" and prophesy smooth things, or did the transport people refuse to bring up the supplies?

